# Effect of bioceramic root canal sealers on the bond strength of fiber posts cemented with resin cements

**DOI:** 10.1590/0103-6440202204529

**Published:** 2022-04-29

**Authors:** Rafael Nesello, Isadora Ames Silva, Igor Abreu De Bem, Karolina Bischoff, Matheus Albino Souza, Marcus Vinícius Reis Só, Ricardo Abreu Da Rosa

**Affiliations:** 1 Conservative Dentistry Department, School of Dentistry, Federal University of Rio Grande do Sul - UFRGS, Porto Alegre, Brazil;; 2 Department of Endodontics, University of Passo Fundo, Passo Fundo, Rs, Brazil;

**Keywords:** Bioceramic, calcium silicate-based sealer, fiber post, push-out bond strength, resin cement

## Abstract

This study aimed to evaluate the influence of calcium silicate-based sealers on the bond strength of fiber posts using conventional and self-adhesive resin cement. Sixty single-rooted teeth were selected. The canals were prepared with a reciprocating instrument 40.06. The roots were randomly distributed in six groups (n = 10) according to the strategies for root canal filling and fiber posts cementation: AH Plus/RelyX ARC; AH Plus/RelyX U200; Bio C Sealer/RelyX ARC; Bio C Sealer/RealyX U200; Sealer Plus BC/RelyX ARC; and Sealer Plus BC/RelyX U200. The roots were transversally sectioned, and one slice per post-third was obtained. The push-out test was performed at a crosshead speed of 1mm/min. The failure patterns were described after assessment with a stereomicroscope with a 10× magnification. Bond strength was calculated and analyzed using the ANOVA and Tukey test. AH Plus did not influence the bond strength of fiber posts cemented with conventional (RelyX ARC) or with self-adhesive resin cement (RelyX U200). The lowest bond strength values were obtained when calcium silicate-based sealers were associated with conventional resin cement (Bio C Sealer/RelyX ARC and Sealer Plus BC/RelyX ARC). Except for Sealer Plus BC/RelyX ARC, all groups presented lower bond strength at the apical portion compared to the cervical portion of the post. Adhesive failures between cement and post and cement and dentin were predominant (55.3%). Calcium silicate-based sealers decreased the bond strength of fiber posts cemented with conventional resin cement.

## Resumo

Este estudo teve como objetivo avaliar a influência de cimentos à base de silicato de cálcio na resistência de união de pinos de fibra utilizando cimentos resinosos convencionais e autoadesivos. Sessenta dentes humanos monorradiculares foram selecionados. Os canais foram preparados um instrumento reciprocante 40.06. As raízes foram distribuídas aleatoriamente em seis grupos (n = 10) de acordo com as estratégias de obturação do canal e cimentação dos pinos de fibra: AH Plus/RelyX ARC; AH Plus/RelyX U200; Bio C Sealer/RelyX ARC; Bio C Sealer/RealyX U200; Sealer Plus BC/RelyX ARC; e Sealer Plus BC/RelyX U200. As raízes foram seccionadas transversalmente e foi obtido um espécime por terço. O teste de push-out foi realizado em uma velocidade de 1mm/min. Os padrões de falha foram descritos após avaliação em estereomicroscópio com aumento de 10×. A resistência de união foi calculada e analisada por ANOVA e teste de Tukey. AH Plus não influenciou na resistência de união dos pinos de fibra cimentados com cimento resinoso convencional (RelyX ARC) ou autoadesivo (RelyX U200) (p > 0,05). Os menores valores de resistência de união foram obtidos quando os cimentos à base de silicato de cálcio foram associados ao cimento resinoso convencional (Bio C Sealer/RelyX ARC e Sealer Plus BC/RelyX ARC) (p < 0,05). Com exceção do Sealer Plus BC/RelyX ARC, todos os grupos apresentaram menor resistência de união na porção apical em comparação com a porção cervical do pino (p < 0,05). Falhas adesivas entre cimento e pino e cimento e dentina foram predominantes (55,3%). Cimentos a base de silicato de cálcio diminuíram a resistência de união de pinos de fibra cimentados com cimento resinoso convencional.

## Introduction

The restoration of endodontically treated teeth is complex when the dental crown is widely compromised. In these cases, the root canal treatment is essential to permit the cementation of an intraradicular post to retain the restoration. Fiber posts have been an alternative to cast posts and cores, mainly because their elastic moduli close to dentin, which leads to a more uniform stress distribution along the root ^1^. Moreover, it presents favorable esthetic features and promising clinical longevity of restorations retained by fiber posts ^2^.

Some endodontic aspects must be considered when fiber posts will be cemented. The irrigant used during root canal preparation, the sealer used for canal obturation, the time elapsed between canal obturation and post cementation, and the protocol applied for post space cleaning play an essential role in the bond strength of fiber posts to root dentin. The composition of the endodontic sealer or the difficulty in removing it from root canal walls or dentinal tubules can jeopardize the adhesion of fiber posts to root dentin. The negative impact of eugenol-based sealers on fiber posts' bond strength is well established, mainly when the cementation is performed immediately after the endodontic treatment ^3^ ^,^ ^4^. On the other hand, epoxy resin-based sealers have been indicated as the gold standard sealer for root canal obturation previously to post cementation because of their physicochemical and biological properties ^3^.

Calcium silicate-based sealers (i.e., bioceramic sealers) were proposed in Endodontics for root canal obturation. These sealers have alumina, zirconia, bioactive glass, glass-ceramic, and hydroxyapatite ^5^. Bioceramic sealers present high pH, antibacterial activity, and biocompatibility ^5^. During their setting reaction, they can react with the root dentin and induce hydroxyapatite formation ^6^. In Brazil, two commercial brands of calcium silicate-based sealers are available. Sealer Plus BC (MK Life, Porto Alegre, RS, Brazil) presents a pH of around 12, releases calcium ions for at least one week. It presents good flow, a 3.6 mm/Al of radiopacity, but high solubility after in vitro investigations ^7^. Bio-C Sealer (Angelus, Londrina, PR, Brazil) has higher radiopacity (5.5 mm/Al) than Sealer Plus BC but also presents high solubility ^5^. Additionally, it favors the expression of osteoblastic markers and biomineralization when in contact with connective tissues ^5^. 

Several strategies for fiber post cementation have been investigated ^8^. Resin cement can be classified according to the adhesive approach. The conventional resin cement must be used combined with adhesive systems, while the self-adhesive resin cement dispenses the pretreatment of root dentin with phosphoric acid and the use of adhesives ^8^. 

Therefore, the main advantage of using self-adhesive resin cement is the simplification of the clinical steps becoming the operatory technique less operator dependent. RelyX ARC (3M ESPE, Seefeld, Germany) is a dual-cure conventional resin cement that requires an adhesive system. RelyX U200 (3M ESPE) is a self-adhesive resin cement more tolerant to dentin humidity with a simplified technique of use ^8^. Both types of cement present excellent laboratory and clinical results ^8^ ^,^ ^9^. 

Considering the current literature, the evidence of the impact of calcium silicate-based sealers on fiber posts' bond strength is scarce. Thus, this study aimed to investigate the bond strength of fiber posts cemented with conventional or self-adhesive resin cement after root canal obturation with two bioceramic sealers. The null hypothesis is that the bioceramic sealers would not influence the bond strength values of fiber posts cemented with conventional or self-adhesive resin cement.

## Material and Methods

This study was approved by the Research Ethics Committee (CAAE:*26897519.3.0000.5347*) of the UFRGS (Federal University of Rio Grande do Sul, Porto Alegre, Brazil).

### Sample size calculation and tooth selection

Sample size calculation was performed using G*Power v.3.1 for Mac (Heinrich Heine, University of Dusseldorf, Dusseldorf, Germany) and selecting the ANOVA test. All data were based on a previous study ^5^. A standard error deviation of 0.92, the minimum difference between treatment means of 1.65, an alpha-type error of 0.05, and a beta power of 0.8 were stipulated. Thus, sixty single-rooted premolars were used in this study. Only roots with cervical diameters of 5 ± 0.3 mm in the mesiodistal direction and 6 ± 0.3 mm in the buccal-palatal direction were included. Teeth less than 15 mm in length were excluded. Periapical radiographs were taken to confirm one root canal's presence and exclude incomplete root formation, root resorption, external cracks, and a coronal root canal diameter greater than 2 mm, as measured with a digital caliper (Starrett 727; Starrett, Itu, SP, Brazil). All teeth were stored in a 0.9% saline solution at 4°C until use. The roots were transversally sectioned using a diamond disc (Komet, Lemgo, Germany) underwater irrigation to obtain specimens with 15 mm in length.

### Root canal preparation and filling

Working length (WL) was established with a size 10 K-file (Dentsply Maillefer, Ballaigues, Switzerland) and was set at 1 mm from the apex. Root canals were prepared with the 40.06 X1 Blue file (MK Life) up to the full WL in a reciprocation motion using a VDW Silver Motor (VDW, Munich, Germany), operating with the 'Reciproc ALL' program (300 rpm). Irrigation procedures were performed using plastic syringes (Ultradent Products, South Jordan, USA) and 30-G needles (NaviTip, Ultradent Products). All root canals were irrigated with 20 mL of 2.5% sodium hypochlorite 20 mL (Farmácia Marcela, Porto Alegre, RS, Brazil) during the canal preparation. Next, the canals were irrigated with 5 mL of 17% ethylene diamine tetra-acetic acid (EDTA) (Farmácia Marcela). Finally, final irrigation with 5 mL of 0.9% of saline solution (Farmácia Marcela) was performed.

The roots were then divided into three groups (N=20 per group) according to the endodontic sealer: one epoxy resin-based sealer (AH Plus; Dentsply Maillefer) and two bioceramic sealers (Bio-C Sealer and Sealer Plus BC).Table 1 shows the materials used, the manufacturer and their chemical composition.

The canals of specimens from the AH Plus group were dried entirely with paper points size #40 (MK Life). In the groups Bio-C Sealer and Sealer Plus BC (i.e., calcium silicate-based sealers), the saline solution was partially removed from the canal with capillary tips (Angelus) to maintain the moisture into the canal and guarantee the setting reaction of the bioceramic sealers.

AH Plus was handled according to the manufactures' instructions and inserted into the canals to 1 mm short of the WL using a 400-rpm Lentulo spiral (Dentsply Maillefer) for 5 seconds. Bio-C Sealer and Sealer Plus BC were delivered into the canals using the special needles of both systems up to the sealer became visible at the cervical opening. The canals were filled with the single cone technique using 40.06 gutta-percha cones (Dentsply Meillefer) were positioned in the WL. Next, the excess of root filling was removed, and vertical compaction was performed using the Berger Pluggers (Angelus). The canal opening and remaining dentin were conditioned with phosphoric acid 37% for 30s (Condac 37; FGM, Joinvile, SC, Brazil), rinsed, and dried. Next, these structures were treated with a multiple-bottle total-etch adhesive system (ScotchBond Multi-Purpose; 3M ESPE) as recommended by the manufacturer using a microbrush (Cavibrush, FGM). Finally, the specimens were restored with Filtek Z350 composite resin (3M ESPE). All roots were kept in 100% humidity at 37°C for one week to allow the sealers to set completely.

**Table 1:** List of materials with brands, batch number and chemical composition.

	Manufacturer/Batch number	Composition
AH Plus	Dentsply Maillefer, Ballaigues, Switzerland; Batch number: 2103001129	Paste A: Bisphenol-A epoxy resin, Bisphenol-F epoxy resin, calcium tungstate, zirconium dioxide, sílica, and iron oxide pigments
		Paste B: Dibenzyldiamnine, aminoadamantane, tricyclodecane-diamine, calcium tungstate, zirconium dioxide, silica, and silicone oil.
Bio-C Sealer	Angelus, Londrina, PR, Brazil; Batch number: 56929	Calcium Silicate, calcium aluminate, calcium oxide, zirconium oxide, iron oxide, silicon dioxide and dispersing agent.
Sealer Plus BC	MK Life, Porto Alegre, RS, Brazil; Batch number: 27082019	Calcium silicate, zirconium oxide, tri-calcium silicate, calcium hidroxide, propilenoglycol.
Scotch Bond Multipurpose	3M ESPE, St.Paul, MN, USA; Batch number: NA50246	Primer: Aqueus solution of HEMA and polyalcenoic acid copolymers.
		Adhesive: Solution of BIS-GMA and HEMA and combination of amines.
RelyX ARC	3M ESPE, Seefeld, Germany; Batch number: 1908600402	Paste A: Silane-treated ceramic, TEGDMA, BisGMA, silane-treated silica, functionalized dimethacrylate polymer, triphenylantimony.
		Paste B: Silane-treated ceramic, TEGDMA, BisGMA, silane-treated silica, functionalized dimethacrylate polymer, 2-benzotriazolyl-4-methylphenol, benzoyl peroxide.
RelyX U200	3M ESPE, Seefeld, Germany; Batch number: 7331349	Multifunctional phosphoric acid methacrylates, dimethacrylates, acetate, initiator/ stabilizer, powdered glass, silica, substituted pyrimidine, calcium hydroxide, peroxide compound, pigments.

### Experimental groups and cementation procedures

Initially, the canals were re-accessed using #1014 diamond burs (KG Sorensen, Barueri, SP, Brazil) coupled to a high-speed motor (KaVo Dental, Biberach, Germany) under water-cooling. The resin composite was removed, and the root canal filling was partially removed using sizes 3 and 4 Gates Glidden drills (Dentsply Maillefer), keeping 4 mm of gutta-percha at the apical third. Periapical radiographs confirmed the complete filling removal. Next, the post space was prepared 10mm deep to receive size 0.5 Exacto fiber post using the correspondent bur of the system (Angelus). The roots were embedded in chemically cured acrylic resin blocks (Dencrilay, Dencril, Pirassununga, SP, Brazil) as described for ^10^.

The post-space cleaning was performed with 5 mL of 5% NaOCl, followed by 5 mL of 17% EDTA for sixty seconds each. Both irrigation protocols used disposable 5 mL syringes (Ultradent) and 30-G needles (NaviTip, Ultradent). The post space was dried using size 80 paper points (Dentsply) ^11^. Fiber posts were cleaned with 70% ethyl alcohol (Mega Química Ind. Comércio Ltda, Pederneiras, Brazil) and coated with silane-based primer (Prosil; Angelus).

The groups, previously formed based on the endodontic sealer, were subdivided according to the cement used for post cementation: conventional (RelyX ARC) or self-adhesive (RelyX U200) resin cement. Thus, the experimental groups (n=10) were: AH Plus/RelyX ARC; AH Plus/RelyX U200; Bio C Sealer/RelyX ARC; Bio C Sealer/RealyX U200; Sealer Plus BC/RelyX ARC; and Sealer Plus BC/RelyX U200.

In those groups that used conventional resin cement (RelyX ARC), the canal walls were etched with 37% phosphoric acid for 15 seconds, washed with distilled water for 20 seconds, dried with 80 paper points, and then the Scotch Bond Multipurpose adhesive (3M ESPE) system was used. The primer was actively applied for 10 seconds using a microbrush (KG Sorensen), and then an air jet was applied for 10 seconds. The adhesive was also actively applied for 10 seconds in the root canal walls with a microbrush and light cured. RelyX ARC was inserted into the root canal using Automix tips, and the fiber post was positioned. The self-adhesive resin cement (RelyX U200) was also mixed and inserted into the canals using automix tips but with no pre-treatment of root dentin. Both types of cement were light-cured for 40 seconds using a previously calibrated LED light-curing unit (Radii Cal; SDI, Bayswater, Australia) with a power of 600mV/cm^2^. A single operator performed all procedures (R.N.). The specimens were stored in 100% humidity for 48 hours at 37°C.

### Push-out test and failure pattern analysis

The roots were transversally sectioned using a diamond disc under water cooling and a cutting machine (Extec Labcut 1010, Enfield, USA). Three slices per specimen (thickness: 2 ± 0.3 mm) were obtained, one for each post third. Each slice was positioned on a metallic device with a central opening (Ø= 3 mm) larger than the canal diameter. A metallic cylinder with a flat tip (Ø tip = 0.8 mm) induced a load on the post in an apical to coronal direction without any pressure to the cement and dentin for push-out testing.

The push-out test was made in a universal test machine (Emic DL-2000; Emic, São José dos Pinhais, Brazil) at a 1 mm/min speed. The bond strength values (σ) in MPa were obtained as follow: σ=F/A, where F = load for rupture (N) and A = bonded area (mm^2^). To determine the bonded interface area, a formula was used: A = 2 × π × g(R1 + R2), where π = 3.14, g = slant height, R1 = smaller base radius, R2 = larger base radius. The following calculation was used to determine the slant height: g^2^ = (h^2^ + [R2 - R1]^2^), where h = section height. R1 and R2 were obtained by measuring the internal diameters of the smaller and larger base, respectively, which corresponded to the internal diameter between the root canal walls. The diameters and height were measured using a digital caliper ^10^. One blinded operator (I.A.B.) performed the tests, and the other (I.A.S.) performed the measurements.

Each slice was analyzed in a stereomicroscope (StereoDiscovery V20, Carl-Zeiss, Gottingen, Germany) with a 10× magnification by two researchers (I.A.B. and I.A.S). If there was no agreement between them, a third researcher (R.A.R.) assessed the samples. So, the failure patterns were classified as adhesive at the cement/dentin interface, adhesive at cement/post interface, cohesive for the dentin, cohesive for the cement, cohesive for the post, and mixed. Representative images of the failure patterns were obtained with scanning electron microscopy (SEM) at 100×, 150×, and 500× magnification (Zeiss EVO MA10, Carl-Zeiss). Specimens with cohesive failures were excluded from the bond strength calculation since this failure pattern does not represent a real push-out bond strength ^12^. 

### Statistical Analysis

Results were statistically analyzed by using SPSS for Windows software (SPSS Inc.). The Shapiro-Wilk test confirmed a normal distribution of the data. The bond strength values were compared using one-way ANOVA and Tukey post-hoc tests. The level of significance was set at 5%.

Results

Table 2 presents the mean bond strength values of the experimental groups according to the endodontic sealer used for root canal filling and the resin cement used for fiber post cementation. The intergroup analysis showed that the calcium silicate-based sealers decreased the mean bond strength values of fiber posts cemented with RelyX ARC (p < 0.05). The lowest mean bond strength values were observed in Bio-C Sealer/RelyX ARC and Sealer Plus BC/RelyX ARC groups (p < 0.05). AH Plus did not influence the bond strength of fiber posts cemented with RelyX ARC and RelyX U200 (p < 0.05).

In the cervical portion, Sealer Plus BC/RelyX ARC and Sealer Plus BC/RelyX U200 presented the lowest bond strength values (p < 0.05). In the middle third, AH Plus/RelyX U200 showed the highest values (p < 0.05) and the Sealer Plus BC/RelyX ARC the lowest (p < 0.05). Finally, except for Sealer Plus BC/RelyX U200 (p < 0.05), all groups presented low bond strength values in the apical third. 

The intragroup analysis showed higher bond strength values at the cervical portion of the post compared with the apical region in AH Plus/RelyX ARC, Bio-C Sealer/RelyX ARC and Bio-C Sealer/RelyX U200 groups (p < 0.05). Sealer Plus BC/RelyX ARC and Sealer Plus BC/RelyX U200 presented lower bond strength at cervical than at the apical portion of the post (p < 0.05). 

**Table 2:** Mean values of bond strength (standard deviations, in MPa) for the experimental groups after push-out test.

	AH Plus/RelyX ARC	AH Plus/Rely X U200	Bio-C Sealer/RelyX ARC	Bio-C Sealer/RelyX U200	Sealer Plus BC/RelyX ARC	Sealer Plus BC/RelyX U200
Cervical	11.6 ± 3.5 Aa	12.9 ± 4.9 Aab	8.2 ± 1.9 Ba	14.3 ± 4.0 Aa	4.1 ± 2.1 Ca	5.2 ± 1.5 Cb
Middle	13.3 ± 3.0 Ba	17.5 ± 4.0 Aa	8.7 ± 0.6 Ca	11.2 ± 2.7 BCa	3.1 ± 1.8 Da	8.4 ± 4.4 Cb
Apical	5.6 ± 4.4 Ab	8.1 ± 5.7 Ab	1.4 ± 0.4 Ab	6.5 ± 2.7 Ab	4.9 ± 3.1 Aa	16.7 ± 9.6 Ba
Mean	10.2 ± 4.9 A	12.8 ± 6.1 A	6.2 ± 3.7 B	10.6 ± 4.5 A	4.0 ± 2.4 B	10.1 ± 7.7 A

Different capital and small letters in the line and the column, respectively, denote differences after one-way ANOVA and Tukey’s post hoc tests (P < 0.05).

**Figure 1:**
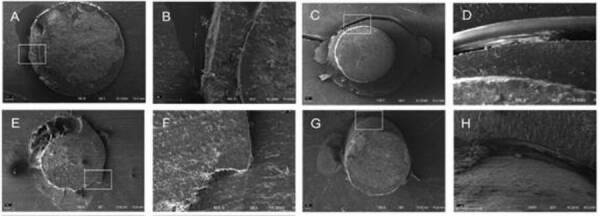
Representative images of some failure patterns analyzed using SEM after the push-out bond strength test. A - Mixed failure in the middle third of AH Plus/RelyX U200. Association of adhesive failure at cement/post, dentin/cement and cohesive of the sealer (150×); B - Magnification of the white square in the image A showing the interfaces dentin-cement-post (500×); C - Adhesive failure in the cervical third of Bio-C Sealer/RelyX ARC (100×); D - Magnification of the white square in the image C showing the dentin-cement interface (500×); E - Mixed failure in the apical third of Bio-C Sealer/RelyX ARC. Association of adhesive failure at cement/post, dentin/cement and cohesive of the sealer (100×); F - Magnification of the white square in the image G showing the failures patterns (500×); G - Adhesive failure in the middle third of Sealer Plus/BC/RelyX U200 (100×); H - Magnification of the white square in the image E showing the dentin-cement interface (500×).

Table 3 presents the failure patterns after the push-out test. The most prevalent failure was adhesive (53.3%), 37.8% adhesive at cement/dentin, and 17.5% adhesive at cement/post interface. Cohesive failures occurred in 24% of the slices, mainly in groups that associated calcium silicate-based sealers and the self-adhesive cement. Figure 1 show some SEM images with the failure patterns (100×, 150×, and 500× magnification).

**Table 3:** Failure mode distribution in each experimental group after push-out test.

Groups	ACD	ACP	M	CD	CC	CP
AH Plus/RelyX ARC	8	15	8	24	3	2
AH Plus/RelyX U200	28	10	8	12	2	-
Bio-C Sealer/RelyX ARC	20	21	6	12	1	-
Bio-C Sealer/RelyX U200	13	10	-	35	2	-
Sealer Plus BC/RelyX ARC	38	3	5	14	-	-
Sealer Plus BC/RelyX U200	29	4	1	26	-	-
Total	136 (37.8%)	63 (17.5%)	28 (7.8%)	123 (34.1%)	8 (2.2%)	2 (0.6%)

ACD = Adhesive failure at cement/dentin interface; ACP = Adhesive failure at cement/post interface; M = Mixed; CD = Cohesive of the dentin; CC = Cohesive of the cement; CP = Cohesive of the post.

## Discussion

Intraradicular posts stabilize the restoration of endodontically treated teeth with extensive crown destruction. Fiber posts have aesthetic and biomechanical characteristics like dentin that reduce the risk of root fractures. Some aspects can interfere with the longevity of the fiber posts, such as the endodontic sealer used to obturate the root canals ^3^ ^,^ ^13^ and the resin cement used to cement these posts ^9^. So, the present study aimed to investigate the bond strength of fiber posts cemented with conventional or self-adhesive resin cement after root canal obturation with two bioceramic sealers. The null hypothesis was rejected because the bioceramic sealers decreased the bond strength values of fiber posts cemented with conventional resin cement. 

The association of bioceramic sealers and conventional resin cement (Bio-C Sealer/RelyX ARC e Sealer Plus BC/RelyX ARC) presented lower bond strength values than the other groups. This study confirmed previous reports in which calcium silicate-based cement before cementing fiber posts with conventional cement can reduce their bond strength to the root dentin ^14^ ^,^ ^15^. After post cementation with RelyX ARC, a previous study ^15^ reported bond strength values twice higher when the canals were filled with AH Plus than bioceramic sealer (Endosequence BC; Brasseler, USA. These results can be explained by the affinity between the components of epoxy resin-based cement and the conventional or self-adhesive resin cement used to cement fiber posts ^16^. 

In this study, Bio-C Sealer/RelyX ARC and Sealer Plus BC/RelyX ARC groups showed decreased bond strength of 40% to 60%, respectively, compared to the AH Plus/RelyX ARC group. Remnants of bioceramic sealer in the entrance of the dentinal tubules can be the main responsible for this result ^14^ ^,^ ^15^. In addition, tag-like structures consisting of either sealer itself or hydroxyapatite crystals may have been generated, suggesting intratubular precipitation ^17^. These precipitations rich in calcium and phosphate, due to the high alkaline pH, could decrease the effectiveness of etching with phosphoric acid and hinder the formation of a hybrid layer by resinous tags of conventional resin cement. Finally, the more significant number of steps required to cement fiber posts using acid conditioning and a three-step adhesive system makes the technique more critical and operator-dependent. 

The protocol used for cleaning the post space must be considered regardless of the endodontic sealer used for canal obturation ^11^ ^,^ ^12^. Epoxy resin-based sealers are more easily removed from the root canal walls than other types of cement ^18^. Remnants of sealer can block dentin tubules, decreasing dentin wettability, permeability, and reactivity, affecting the adhesive interface and bond strength ^19^. Besides, the filling removal depends on the chemical compound of the irrigant used for post space cleaning and the physical phenomenon of sonic or ultrasonic activation of the irritant ^11^ ^,^ ^12^. Best results were observed when the post space was irrigated with 5 mL of 5% NaOCl followed by 5 mL of 17% EDTA ^11^. On the other hand, the ultrasonic activation for 20 seconds of saline or NaOCl 2.5% presented better results than 17% EDTA, QMix, and Smear Clear ^12^. In both cases, the fiber posts were cemented with self-adhesive resin cement. In this study, the post space was irrigated with 5 mL of 5% NaOCl followed by 5 mL of 17% EDTA for all experimental groups.

AH Plus/RelyX ARC, Bio-C Sealer/RelyX ARC and Bio-C Sealer/RelyX U200 groups presented higher bond strength at the cervical portion of the post compared with the apical region. The density and diameter of dentin tubules are higher in the cervical than in the apical third of the root ^20^. These characteristics make the root dentin conditioning and adhesive penetration into dentinal tubules difficult for conventional resin cements (i.e. RelyX ARC). The porous regions into the hybrid layer leave spaces around the collagen fibrils, generating lower values of bond strength from coronal to apical portions of the canal ^20^. Also, for both cements, a negative aspect of bond strength into the root canal is the limited ability of conventional and self-adhesive resin cement to dissipate the stress generated during polymerization shrinkage ^20^. The cavity configuration factor (C-Factor) is related to the ratio between the adhered area and the free area, which is extremely high within the root canal. It causes gaps at the adhesive interface between the cement and the post and between the cement and the dentin, affecting the bond strength and leading to adhesive failures ^10^ ^,^ ^20^. Finally, as more apical the post space level, the more difficult it is to control the adhesive technique (i.e. for conventional resin cements), visualization, access and light-curing ^20^. All these factors contribute to an inadequate polymerization in the apical region of the post. 

This study showed a predominance of adhesive failures after the push-out test (55.3%), with 37.8% at cement/dentin interface and 17.5% at cement/post interface. Similar results were observed in previous studies ^15^ ^,^ ^21^. As previously mentioned, the high C-Factor observed in the root canal, the difficulty in controlling dentin's humidity, and the difficulty of light-curing the adhesive system and resin cement into the root canal, especially in the apical area, contribute to failures of this nature ^20^. In 24% of the samples, cohesive dentin failures were observed, especially in groups where bioceramic sealers (Bio-C Sealer and Sealer Plus BC) were associated with self-adhesive cement (RelyX U200). This result probably occurred due to the chemical interaction between bioceramic sealers and root dentin and between bioceramic sealer and self-adhesive resin cement that chemically bonds to dentin and the remainder of calcium silicate-based filling cement.

It should be noted that the cohesive failures were excluded from the bond strength calculation since they do not correspond effectively to the bonding force between the restored material and the dentin ^10^. The push-out test was used in this study because it can create a uniform stress concentration area at the adhesive interface ^22^. One of the limitations of this study is that the laboratorial test was carried out under controlled conditions and should not be used alone to indicate or contraindicate clinical decisions. Besides, low intermittent loads and temperature changes (i.e cyclic and thermo cycling, respectively) could better simulate the function of endodontically treated teeth restored with fiber posts. However, this study was not designed to answer these questions. Further studies must investigate the aging of the specimens. 

Based on the results of this laboratory investigation, the bioceramic sealers (Bio-C Sealer and Sealer Plus BC) decreased the bond strength values of fiber posts cemented with conventional resin cement (RelyX ARC) but not with self-adhesive resin cement (RelyX U200). The use of calcium silicate-based sealers should be avoided previously fiber posts cementation with conventional resin cement. 
